# Supplementary material for: Cavity-enhanced photon indistinguishability at room temperature and telecom wavelengths
Source: Nat Commun. 2024 May 11;15:3989. doi: 10.1038/s41467-024-48119-1 (PMC11088649; doi:10.1038/s41467-024-48119-1)
Supplement: Supplementary file 1 — Supplementary Information [file 41467_2024_48119_MOESM1_ESM.pdf]

Supplementary Information: Cavity-enhanced photon indistinguishability at room temperature and telecom wavelengths

Lukas Husel,[1,*] Julian Trapp,[1,*] Johannes Scherzer,[1] Xiaojian Wu,[2] Peng Wang,[2] Jacob Fortner,[2] Manuel Nutz,[3] Thomas Hümmer,[3] Borislav Polovnikov,[1] Michael Förg,[3] David Hunger,[4,5] YuHuang Wang,[2] and Alexander Högele[1,6]

[1] *Fakultät für Physik, Munich Quantum Center, and Center for NanoScience (CeNS), Ludwig-Maximilians-Universität München, Geschwister-Scholl-Platz 1, 80539 München, Germany*
[2] *Department of Chemistry and Biochemistry, University of Maryland, Maryland, USA*
[3] *Qlibri GmbH, Maistr. 67, 80337 München, Germany*
[4] *Physikalisches Institut, Karlsruhe Institute of Technology, Karlsruhe, Germany*
[5] *Institute for Quantum Materials and Technologies (IQMT), Karlsruhe Institute of Technology (KIT), Herrmann-von-Helmholtz Platz 1, 76344 Eggenstein-Leopoldshafen, Germany*
[6] *Munich Center for Quantum Science and Technology (MCQST), Schellingstr. 4, 80799 München, Germany*

(Dated: January 18, 2024)

SUPPLEMENTARY NOTE 1 - REGIME OF INCOHERENT GOOD NTD-CAVITY COUPLING

In the regime of incoherent good cavity coupling, $2g \ll \gamma + \gamma^* + \kappa$ and $\kappa < \gamma + \gamma^*$ holds [1] , where g is the light-matter coupling strength, γ is the population decay rate, κ is the cavity linewidth and γ^* is the pure dephasing rate. $\gamma = \gamma_r + \gamma_{nr}$ is the sum of radiative and nonradiative decay rates γ_r and γ_{nr}, respectively. We determined $\kappa = 35.4 \pm 0.1$ μeV for the lowest accessible longitudinal mode order, and $\gamma^* = 8 \pm 2$ meV from the cavity length sweep in Fig. 2b of the main text. As pointed out ibidem, our two-photon interference and PL lifetime measurements indicate a biexponential NTD population decay with fast and slow timescales $\tau_{\text{fast}} = 2$ ps and $\tau_{\text{slow}} = 91$ ps, respectively, corresponding to $\gamma_{\text{fast}} = 330$ μeV and $\gamma_{\text{slow}} = 7.3$ μeV. Obviously, $\kappa < \gamma + \gamma^*$ holds in our system for both $\gamma = \gamma_{\text{fast}}$ and $\gamma = \gamma_{\text{slow}}$.

We estimate the light-matter coupling strength using

$$g = \sqrt{3\lambda^2 c/(8\pi n^3 V_{\text{c}} \tau_{\text{rad}})}, \tag{1}$$

with the speed of light c, the wavelength λ, the cavity mode volume V_{c}, the refractive index n and the radiative lifetime τ_{rad} [2]. For our cavity, we calculated $V_{\text{c}} = 8.2$ μm^3 for the lowest accessible mode order. We use $n = 1$ in our estimate of the light-matter coupling strength, neglecting that the NTDs are placed on a polystyrene spacer with refractive index $n = 1.57$. This results in an upper bound for g. For the type of NTDs investigated in this work, τ_{rad} was found to range between 1 and 15 ns [3, 4]. Varying τ_{rad} within $1 - 15$ ns, we expect g to range between 17 and 64 μeV. With the results above, we conclude that $2g \ll \gamma + \gamma^* + \kappa$ holds in our system, consistent with the regime of incoherent good cavity coupling.

SUPPLEMENTARY NOTE 2 - MODEL FOR NTD-CAVITY COUPLING DYNAMICS

We first consider a two-level emitter coupled to an optical cavity. This setting was studied in Ref. [1], where the time-dependent density operator $\hat{\rho}(t)$ of the coupled emitter-cavity system was obtained from a Lindblad master equation in Markovian approximation. In the regime of incoherent cavity coupling, the influence of the density operator coherences on the system dynamics was shown to be negligible. The coupled system is therefore fully described by the populations of cavity and emitter, which exchange photons at a rate R given by [5]:

$$R = 4g^2/(\kappa + \gamma + \gamma^*). \tag{2}$$

As explained in the main text, our two-photon interference and PL lifetime measurements indicate a biexponential NTD population decay, attributed to the presence of an additional dark excitonic reservoir. In order to model the

---

* These authors contributed equally to this work

coupling of such an NTD to a cavity, we first focus on the value of R in our system. Given incoherent cavity coupling, the denominator in Eq. 2 is dominated by the pure dephasing rate γ^*, such that $R \approx 4g^2/\gamma^*$. From the measured value $\gamma^* = 8 \pm 2$ meV and the estimated range for g ($17 - 64$ μeV), we expect R to range between 0.14 and 2.04 μeV.

We now extend the model of Ref. [1] to an NTD exhibiting dark and bright exciton states. The dark state has no effect on the limit of incoherent coupling due to its vanishingly small coupling to the cavity. The bright state, on the other hand, will exchange photons with the cavity at rate R, analogous to a radiative two-level system. Based on these considerations, we describe our experiment with the set of partial differential equations:

$$\frac{\partial \rho_{\mathrm{c}}}{\partial t} = -(\kappa + R)\rho_{\mathrm{c}} + R\rho_{\mathrm{b}} \tag{3}$$

$$\frac{\partial \rho_{\mathrm{d}}}{\partial t} = -\gamma_d \rho_{\mathrm{d}} + \gamma_{\mathrm{bd}}\rho_{\mathrm{b}} - \gamma_{\mathrm{db}}\rho_{\mathrm{d}} \tag{4}$$

$$\frac{\partial \rho_{\mathrm{b}}}{\partial t} = -(\gamma_b + R)\rho_{\mathrm{b}} + R\rho_{\mathrm{c}} - \gamma_{\mathrm{bd}}\rho_{\mathrm{b}} + \gamma_{\mathrm{db}}\rho_{\mathrm{d}} \tag{5}$$

with the populations of the cavity ρ_{c}, dark state ρ_{d} and bright state ρ_{b}. Here, $\gamma_{\mathrm{bd/db}}$ is the population exchange rate between ρ_{b}, and ρ_{d} and $\gamma_{\mathrm{b/d}}$ are the sum of radiative and nonradiative decay rates of ρ_{b} and ρ_{d}.

The parameters $\gamma_{\mathrm{b/d}}$, $\gamma_{\mathrm{bd/db}}$ and $\rho_b(0)$ are free parameters in our model. We choose them such that our model prediction fulfils two criteria. First, the predicted time-dependent PL intensity, which is the cavity population ρ_{c} convoluted with the instrument response function, should agree with the measured time-dependent PL in Fig. 4d of the main text. Second, the biexponential bright state decay, obtained for setting $R = 0$ in Eqns. 3 – 5, should have a short population lifetime $\tau_{\mathrm{short}} = 2$ ps. This is indicated by the two-photon interference measurement in Fig. 4b of the main text, as explained ibidem and in Supplementary Note 5. The result of this parameter adaptation is the solid line in Fig. 4d and agrees well with the measured data.

SUPPLEMENTARY NOTE 3 - REGIME OF LOW PURCELL ENHANCEMENT

A. Purcell factor and PL lifetime

The PL lifetime τ_{pl} of emitters coupled to the cavity is given by $\tau_{\mathrm{pl}} = \tau_{\mathrm{fs}}/(1 + F_{\mathrm{p}}^*)$, with the free-space population lifetime $\tau_{\mathrm{fs}} = 1/\gamma$ and the effective Purcell factor $F_{\mathrm{p}}^* = R/\gamma$ [6]. For the slow population decay component with $1/\gamma = 91$ ps, F_{p}^* ranges between 0.018 and 0.28 for the previously estimated range of R ($0.14 - 2.04$ μeV). For the fast decay component, the expected value for F_{p}^* is even smaller ($4.1 \cdot 10^{-4} - 6.2 \cdot 10^{-3}$). We infer from this result that population lifetime shortening effects due to the cavity coupling are negligible in our system, corresponding to a regime of low Purcell enhancement.

While F_{p}^* quantifies enhancement of the total emitter decay rate by cavity-coupling, the enhancement of the radiative decay rate $\gamma_{\mathrm{rad}} = 1/\tau_{\mathrm{rad}}$ is quantified by $F_{\mathrm{p}} = 3\lambda^3 Q_{\mathrm{eff}}/(4\pi^2 n^3 V_c)$, with the effective Q-factor $Q_{\mathrm{eff}} = (Q_{\mathrm{cav}}^{-1} + Q_{\mathrm{em}}^{-1})^{-1}$ and the Q-factors of cavity Q_{cav} and emitter Q_{em}, respectively. In the limit of incoherent coupling, F_{p} approximates to $F_{\mathrm{p}} \approx R/\gamma_{\mathrm{rad}} \approx 3\lambda^2 c/(4\pi V_c \gamma^*)$ [7]. For our system, we expect $F_{\mathrm{p}} = 1.6$. We note that for $\kappa \gg \gamma + \gamma^*$ (bad cavity regime, different from our experiment), F_{p} is called ideal Purcell factor [8] and evaluates to the original expression given by Purcell [9] $F_{\mathrm{p}} \approx 3\lambda^3 Q_{\mathrm{c}}/(4\pi^2 n^3 V_c) = 4g^2/(\gamma_{\mathrm{rad}}\kappa)$. Finally, we also note that the effective Purcell factor can be expressed as $F_{\mathrm{p}}^* = \eta_Q F_{\mathrm{p}}$, with the quantum yield $\eta_Q = \gamma_r/\gamma$. From the expected range of radiative lifetimes given above, we infer an estimated range of $6 \cdot 10^{-3} - 9 \cdot 10^{-2}$ for η_Q, where we only considered the fast decay component since the majority of the population decays on this timescale (see Supplementary Note 4). The estimated values agree with previously measured NTD quantum yields.

B. Single photon efficiency

The single photon emission efficiency β_{c} gives the probability that a photon is emitted into the spectral window of the cavity linewidth κ, given an initial excitation of the emitter. Each excitation pulse generates a photon in the cavity with probability $\beta_{\mathrm{c}}\eta_{\mathrm{em}}$, where η_{em} is the free space photon emission efficiency at the respective pump power. The rate of photons registered by the detector I_{em} is then given by:

$$I_{\mathrm{em}} = f_{\mathrm{exc}}\eta_{\mathrm{out}}\eta_{\mathrm{sys}}\eta_{\mathrm{em}}\beta_{\mathrm{c}}, \tag{6}$$

where f_{exc} is the repetition rate of the excitation source, η_{out} is the probability for a photon to exit the cavity through the flat mirror, η_{sys} is the combined transmission and detection efficiency of the setup.

The measurement of η_{em} would require excitation near or above the saturation threshold. This in turn requires high excitation powers, which can lead to NTD degradation and limit single photon purity and indistinguishability. Since NTD 1, 2 and 3 were used to benchmark single photon purity and indistinguishability, we refrained from measurements at such excitation powers. However, using the maximum measured value $I_{\mathrm{em}} = 1840 \pm 30$ counts/s for NTD1 and the upper bound $\eta_{\mathrm{em}} = 1$, combined with the measurement of η_{sys} and the value for η_{out} obtained from transfer matrix simulations of the mirror coating, we obtain a lower bound of $\min(\beta_{\mathrm{c}}) = (3.9 \pm 0.1) \cdot 10^{-3}$ for the single photon emission efficiency. The theoretically expected value is calculated from the time-dependent cavity population ρ_{c} as [1]:

$$\beta_{\mathrm{c}} = \kappa \int \rho_{\mathrm{c}}(t)dt. \tag{7}$$

Solving Eqns. 3 – 5 for ρ_c, we obtain an expected value $\beta_{\mathrm{c}} = 6.6 \cdot 10^{-3}$ for the single photon emission efficiency.

In the main text, we compared the above values to the expected upper bound for the emission efficiency for spectrally filtered free-space emission β_{fs}, similar to Ref. [1]. The actual value for this quantity is likely smaller due to the non-unity quantum yield in our system, which we expect to reduce β_{fs} to $\eta_Q\kappa/(\pi\gamma^*)$. This expression is easily understood by noting that β_{fs} gives the probability that a photon is emitted into the spectral window of a filter with bandwidth κ, given an initial excitation of the emitter. Using the estimated range for η_Q given above, we expect that the single-photon emission efficiency in our system outperforms that expected for filtered free space emission by at least a factor of 44. This drastic increase in emission spectral density is a direct consequence of cavity-coupling in the good cavity regime and was found to be quantified by the ideal Purcell factor $F_{\mathrm{p,ideal}} = 3\lambda^3 Q_{\mathrm{c}}/(4\pi^2 n^3 V_c)$ as defined in Supplementary Note 3.A [10], for which we expect $F_{\mathrm{p,ideal}} = 91$ for the parameters given in Supplementary Note 1.

We also use the measured single photon efficiency to confirm the result for the light-matter coupling strength g from Supplementary Note 1. If defined as in Eq. 6, the efficiency is given by $\beta_{\mathrm{c}} = F_{\mathrm{p}}/(F_{\mathrm{p}} + \gamma/\gamma_{\mathrm{rad}})$ [8]. We note that this expression is valid for all regimes of cavity-coupling. From the measured values of slow population decay lifetime and β_{c}, we find an experimental upper bound $\max(\tau_{\mathrm{rad}}) = 35.0 \pm 0.9$ ns for the radiative lifetime, corresponding to $\min(g) = 13.5 \pm 0.3$ μeV. A more realistic value is obtained by also considering the fast population decay component. Since $\tau_{\mathrm{fast}} \ll \tau_{\mathrm{slow}}$, both decay processes contribute to the overall efficiency on different timescales, such that we can approximate $\beta_{\mathrm{c}} \approx \tilde{A}_{\mathrm{fast}} F_{\mathrm{p}}/(F_{\mathrm{p}} + \gamma_{\mathrm{fast}}/\gamma_{\mathrm{rad}}) + \tilde{A}_{\mathrm{slow}} F_{\mathrm{p}}/(F_{\mathrm{p}} + \gamma_{\mathrm{slow}}/\gamma_{\mathrm{rad}})$, with the fractional amplitudes $\tilde{A}_{\mathrm{fast/slow}}$ defined as in Eq. 15. We find $\tau_{\mathrm{rad}} = 12.3 \pm 0.3$ ns for the radiative lifetime, corresponding to $g = 22.8 \pm 0.6$ μeV, in agreement with the result from Supplementary Note 1.

C. Cavity-enhancement of PL intensity

From Eq. 6, we derive a quantitative description of the increase in the PL intensity observed in Fig. 2c of the main text as the cavity length is tuned to the lowest accessible mode order. The single photon emission efficiency is given by $\beta_{\mathrm{c}} = \kappa R/[\kappa R + \gamma(\kappa + R)]$ [5], which in the Purcell regime ($R \ll \kappa$, as is the case for our system) simplifies to $\beta_{\mathrm{c}} \approx R/\gamma/(1 + R/\gamma) = F_{\mathrm{p}}^*/(1 + F_{\mathrm{p}}^*)$. Combined with Eqns. 1 and 2, we find $I_{\mathrm{em}} \propto R \propto V_{\mathrm{c}}^{-1}$ to first order in R, which quantitatively describes the behaviour observed in Fig. 2c. We conclude that in our system, cavity coupling increases the single photon emission efficiency β_{c} via enhancement of light-matter coupling strength.

SUPPLEMENTARY NOTE 4 - TWO-PHOTON INTERFERENCE VISIBILITY

We quantify the indistinguishability of photons emitted by the NTD-cavity system by the two-photon interference visibility v one would obtain in an interferometer with balanced beamsplitters (BSs) and unity classical visibility. To determine v from the experimental correlation histograms, we account both for imbalanced interferometer arms and non-ideal single photon purity of each NTD.

A fiber-based interferometer as in Fig. 4a of the main text was used to perform two-photon interference experiments. The stream of photons entering the interferometer was divided at BS 1 with transmission and reflection T_1 and R_2, respectively, and recombined after a tunable delay at BS 2 with transmission and reflection T_2 and R_2. In our interferometer, the transmission of the delay arm is $\mu < 1$. The delay time equals the excitation pulse separation, and is orders of magnitude larger than the coherence time and population lifetimes in our system. We therefore treat the reduced transmission in the delay arm as an effect of imbalanced transmission and reflection of BS 1, and use the effective values $\tilde{T}_1 = T_1/(T_1 + \mu R_1)$ and $\tilde{R}_1 = \mu R_1/(T_1 + \mu R_1)$ for its transmission and reflection.

Using effective transmission and reflection values, we derive expressions for the integrated peak counts N in the experimental histograms (as shown in Supplementary Fig. 1 for NTD 3) obtained by integrating correlation events in

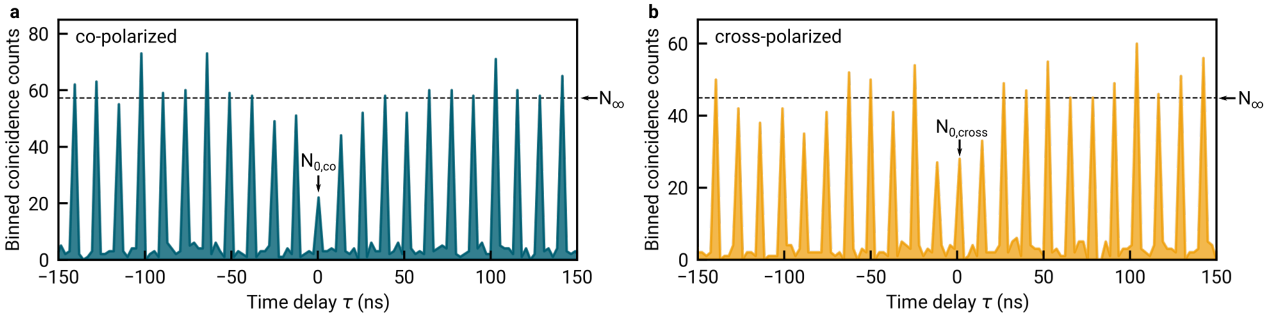

SUPPLEMENTARY FIG. 1. **HOM autocorrelation histograms for NTD3.** HOM correlations for NTD 3 for co-polarized (**a**) and cross-polarized (**b**) interferometer arms with delay of one excitation pulse. Coincidence counts were binned in 2.5 ns time windows. The HOM autocorrelation function in Fig. 4b of the main text was calculated from the displayed data (see the Methods section for details).

a 2.5 ns time window. We extend the calculation of Ref. [11] to obtain N from the intensity autocorrelation between the output ports of BS 2. For large $|\tau|$, we find

$$N_\infty = H\left[\tilde{R}_1\tilde{T}_1(R_2^2+T_2^2)+R_2T_2(\tilde{R}_1{}^2+\tilde{T}_1{}^2)\right], \tag{8}$$

with an integration constant H.

For the height of the peak at $\tau = 0$ for co-polarized interferometer arms (Supplementary Fig. 1a), we find

$$N_{0,co} = H\left[\tilde{R}_1\tilde{T}_1\left[1-2R_2T_2-2R_2T_2(\epsilon_P)^2v\right]+g^{(2)}_{\mathrm{HBT}}(0)R_2T_2(1-2\tilde{R}_1\tilde{T}_1)\right]. \tag{9}$$

In this expression, ϵ_P is the overlap between the polarization modes of the interferometer arms. The height of the central histogram peak for cross-polarization $N_{0,cross}$ (Supplementary Fig. 1b) is obtained by setting $v = 0$ in Eq. 11, which yields

$$N_{0,\mathrm{cross}} = H\left[\tilde{R}_1\tilde{T}_1\left(1-2R_2T_2\right)+g^{(2)}_{\mathrm{HBT}}(0)R_2T_2(1-2\tilde{R}_1\tilde{T}_1)\right]. \tag{10}$$

To determine v for NTD 3, we first extracted the raw visibility $v_{\mathrm{raw}} = 1 - g^{(2)}_{\mathrm{HOM,co}}(0)/g^{(2)}_{\mathrm{HOM,cross}}(0)$ from the data in Fig 4b. Next, we calculated $v_{\mathrm{raw}} = 1 - N_{0,\mathrm{co}}/N_{0,\mathrm{cross}}$ from Eqns. 11 and 12 and solved for v, yielding an expression which depends on v_{raw}, $g^{(2)}_{\mathrm{HBT}}(0)$ and the interferometer parameters $\tilde{T}_1$, $\tilde{R}_1$, T_2, R_2 and ϵ_P. Using experimental values for these quantities ($\tilde{T}_1 = 0.4$, $\tilde{R}_1 = 0.6$, $T_2 = 0.49$, $R_2 = 0.51$ and $\epsilon_P = 0.96$), we finally obtained $v = 0.51 \pm 0.21$ for NTD 3. Based on the experimental values $\tilde{T}_1$, $\tilde{R}_1$, T_2, R_2, ϵ_P and $g^{(2)}_{\mathrm{HBT}}(0)$ for NTD 3, we estimate $g^{(2)}_{\mathrm{HOM,cross}}(0) = N_{0,\mathrm{cross}}/N_\infty = 0.53 \pm 0.04$ for cross-polarized interferometer arms, in good agreement with the experimental value of 0.61 ± 0.12.

For NTD 1, we obtained the raw visibility $v_{\mathrm{raw}} = a$ from the amplitude a of the best-fit to the HOM dip in Fig. 4d of the main text, and calculated v from v_{raw}, $g^{(2)}_{\mathrm{HBT}}(0)$, and the interferometer parameters as described above to obtain $v = 0.65 \pm 0.24$ stated in the main text. The observed asymmetric increase in the visibility towards large positive delays is the result of a degradation-induced decrease in the single photon purity during the measurement. For large interferometer delays, we expect $g^{(2)}_{\mathrm{HOM}}(0) = N_{0,\mathrm{cross}}/N_\infty = 0.64 \pm 0.05$ as an estimate for the offset c in the data of Fig. 4d. This value is smaller than the best-fit value $c = 0.80 \pm 0.08$, which could stem from the degradation observed during the measurement resulting in an overall increase in $g^{(2)}_{\mathrm{HOM}}$. In Supplementary Fig. 2, we show the autocorrelation histograms for interferometer delays of -5, -3, 0, 3 and 5 ps, respectively. The reduction of correlation events at zero time delay τ provides evidence for two-photon interference for NTD 1. Each data point in Fig. 4d of the main text was obtained by taking such histograms and computing $g^{(2)}_{\mathrm{HOM}}(0)$ as described above.

For completeness, we also calculate the values of the HOM autocorrelation function at time delays corresponding to one excitation pulse separation, $\tau = \pm 12.5$ ns. As explained e.g. in the supplement of Ref. [12], $g^{(2)}_{\mathrm{HOM}}$ is smaller than one for these delays. For the respective histogram peak heights, we find

$$N_{12.5} = H\left[\tilde{R}_1\tilde{T}_1T_2^2+R_2T_2(1-2\tilde{R}_1\tilde{T}_1)+g^{(2)}_{\mathrm{HBT}}(0)\tilde{R}_1\tilde{T}_1R_2^2\right] \tag{11}$$

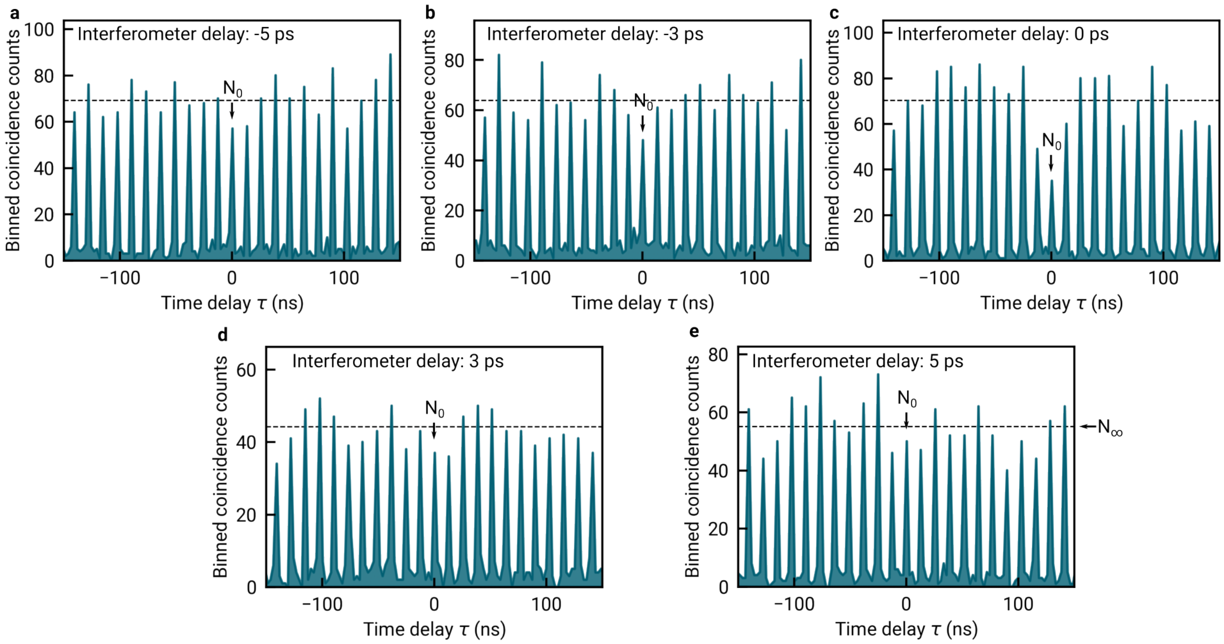

SUPPLEMENTARY FIG. 2. **HOM autocorrelation histograms for NTD 1. a**–**c**, HOM autocorrelation function measured on NTD1 for interferometer delays -5 ps (**a**), -3 ps (**b**), 0 ps (**c**), 3 ps (**d**) and 5 ps (**e**), as used to extract the data points of the HOM dip in Fig. 4c of the main text. The dashed line indicates the mean value of the histogram peaks at time delays $|\tau| > 12.5$ ns, N_∞. An interferometer delay of 0 ps corresponds to separation by exactly one excitation pulse, resulting in maximum probability for two-photon interference and reduced correlation events at zero time delay τ.

and

$$N_{-12.5} = H\left[\tilde{R}_1\tilde{T}_1R_2^2 + R_2T_2(1-2\tilde{R}_1\tilde{T}_1) + g^{(2)}_{\mathrm{HBT}}(0)\tilde{R}_1\tilde{T}_1T_2^2\right]. \tag{12}$$

We note that these expressions are valid for both co- and cross-polarized interferometer arms since the excitation pulse separation greatly exceeds the photon coherence time, leading to vanishing contributions of quantum interference at these time delays. From our measured values of the interferometer parameters, we calculate the expected value $g^{(2)}_{\mathrm{HOM}}(-12.5\text{ ns}) = N_{-12.5}/N_\infty = 0.77 \pm 0.02$, in agreement with the value 0.87 ± 0.13 measured in co-polarized configuration and close to the value 0.58 ± 0.12 measured in cross-polarized configuration. We also expect $g^{(2)}_{\mathrm{HOM}}(12.5\text{ ns}) = N_{12.5}/N_\infty = 0.79 \pm 0.02$, in agreement with the values 0.71 ± 0.12 and 0.75 ± 0.13 measured for co- and cross-polarized interferometer arms, respectively. Overall, these results confirm the good correspondence between our measurements and our theoretical description of the interferometer.

In order to calculate the expected two-photon interference visibility v for cavity-coupled NTDs, we solve Eqns. 3 – 5 for the cavity population ρ_c and evaluate [1]:

$$v = \frac{\int_0^\infty dt\rho_c^2(t)\int_0^\infty d\tau e^{-\Gamma_c\tau}}{\frac{1}{2}\left|\int_0^\infty dt\rho_c(t)\right|^2}. \tag{13}$$

In this equation, $\Gamma_c = \kappa + R$, with $\Gamma_c \approx \kappa$ in our system. With this expression, we find $v = 0.3$ for the theoretically expected visibility without significant dependence on R within the previously estimated range $(0.13 - 2.0\ \mu\text{eV})$.

Finally, to estimate the two-photon interference v for free space NTDs, we set the coupling rate in Eqns. 3 – 5 to zero, $R = 0$, to obtain the free-space bright state decay as:

$$\rho_b(t) = \rho_b(0)\left(A_{fast}e^{-t/\tau_{\mathrm{fast}}} + A_{slow}e^{-t/\tau_{\mathrm{slow}}}\right). \tag{14}$$

In this expression, $A_{fast/slow} \in 0,1$ are amplitude factors which obey $A_{fast} + A_{slow} = 1$. We assume that fast and slow process have visibilites $v_{\mathrm{fast/slow}} = T_2/(2\tau_{\mathrm{fast/slow}})$, where the coherence time is given by the dephasing time as $T_2 \approx 1/\gamma^* = 80$ fs. All simulation results presented in the main text were obtained for a decay with free-space parameters $A_{fast} = 0.92$ and $A_{slow} = 0.08$.

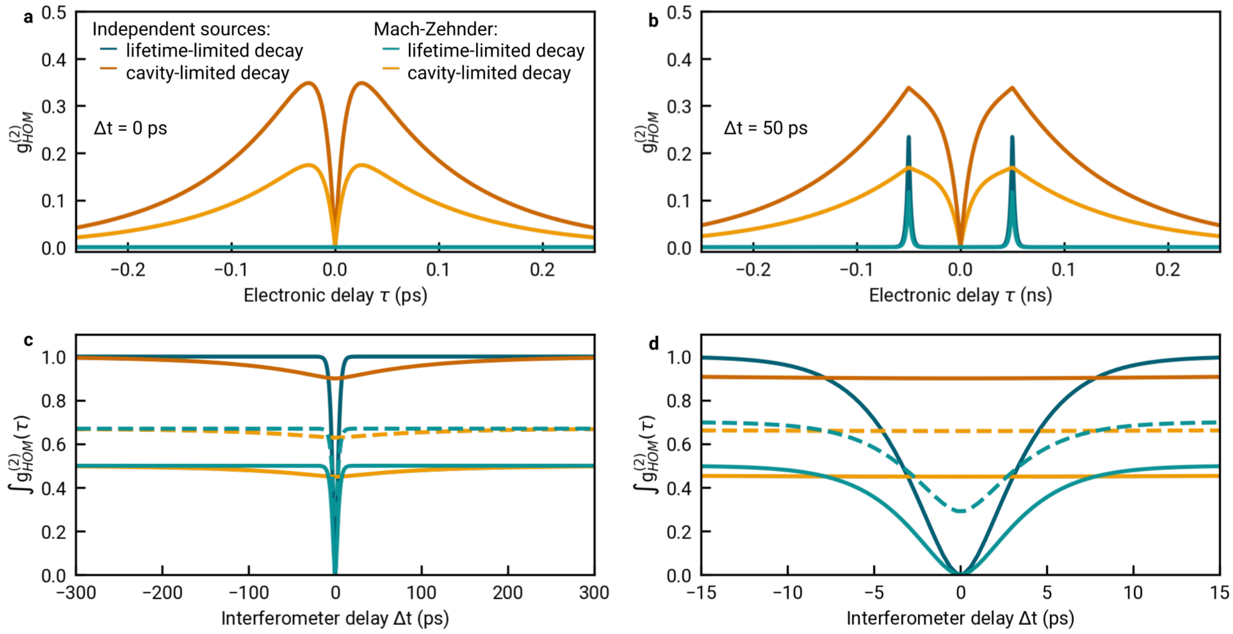

SUPPLEMENTARY FIG. 3. **Two-photon interference timescale.** **a**, **b**, HOM autocorrelation function as a function of electronic delay τ, for interferometer delay $\Delta t = 0$ ps (**a**) and $\Delta t = 50$ ps (**b**). **c**, **d**, Integrated HOM autocorrelation function as a function of interferometer delay Δt. In all panels, the limiting cases of a fast, lifetime limited decay with lifetime 2 ps, and a slow decay with lifetime 100 ps and cavity-limited coherence time 20 ps are considered. Scenarios shown: two independent sources with unity single photon purity probed on a beamsplitter (dark green and orange solid lines); a single source with unity single photon purity probed in a Mach-Zehnder interferometer with 50:50 beamsplitters (blue and yellow solid lines); a single source with non-unity single photon purity (NTD 1) probed in our experimental Mach-Zehnder interferometer with imperfect beamsplitters (blue and orange dashed lines).

The fraction of the population which decays via the fast and slow process, respectively, is quantified by the relative fractional amplitudes $\tilde{A}_{\text{fast/slow}}$ given by [13]:

$$\tilde{A}_{\text{fast/slow}} = \frac{A_{\text{fast/slow}}\tau_{\text{fast/slow}}}{A_{fast}\tau_{\text{fast}} + A_{slow}\tau_{\text{slow}}}. \tag{15}$$

For the emitter dynamics considered in our simulation, we find $\tilde{A}_{fast} = 0.34$ and $\tilde{A}_{slow} = 0.66$. We estimate v as a weighted sum of visibilities for fast and slow process, $v = \tilde{A}_{\text{fast}}v_{\text{fast}} + \tilde{A}_{\text{slow}}v_{\text{slow}}$ to arrive at a vanishingly small free-space visibility $v = 0.003$.

SUPPLEMENTARY NOTE 5 - TWO-PHOTON INTERFERENCE TIMESCALE

As discussed in the main text, we associate the timescale τ_{HOM} in the fit to the HOM dip in Fig. 4d with the emitter population lifetime. To explain this, we consider a monoexponentially decaying emitter with lifetime T_1 and coherence time T_2. Each data point in Fig. 4d is obtained from a correlation histogram as in Fig. 4c of the main text. When probing two independent emitters with unity single photon purity on a beamsplitter, the peak around time delay $\tau = 0$ in such a histogram is described by [14]:

$$g^{(2)}_{\text{HOM}}(\tau) = \frac{1}{4}e^{-|\tau-\Delta t|/T_1} + \frac{1}{4}e^{-|\tau+\Delta t|/T_1} - \frac{1}{2}e^{-|\tau|(2/T_2-1/T_1)-|\tau-\Delta t|/(2T_1)-|\tau+\Delta t|/(2T_1)}. \tag{16}$$

When probing a single emitter in a Mach-Zehnder interferometer with 50:50 beamsplitters, a prefactor of 1/2 has to be included to account for reduced coincidence probability around $\tau = 0$ [12, 15].

We now consider the limiting cases of a lifetime-limited fast decay ($T_1 = 2$ ps, $T_2 = 4$ ps) and a cavity-limited slow decay ($T_1 = 100$ ps, $T_2 = 20$ ps given by the measured cavity lifetime), featured by two independent emitters and

a single emitter probed in a Mach-Zehnder interferometer. We plot the results of Eq. 16 for each of these cases in Supplementary Fig. 3a and b, with interferometer delay $\Delta t = 0$ ps and $\Delta t = 50$ ps, respectively. The lifetime-limited coherence of the fast decay enables two-photon interference, which results in vanishing correlation counts.

As obvious from Eq. 16 and Supplementary Fig. 3a and b, the coherence time T_2 can in principle be probed by varying the electronic delay τ. By contrast, tuning of the interferometer delay changes the photon arrival time at the beamsplitter, such that this measurement probes the emitter population lifetime. In our experiment, the histogram bin size is much larger than the population lifetime and coherence time, and thus the histogram peak at $\tau = 0$ is given by [14]:

$$g^{(2)}_{\mathrm{HOM}}(\tau = 0) = \int_{N_0} g^{(2)}_{\mathrm{HOM}}(\tau) d\tau = \frac{1}{2}\left[1 - \frac{T_2}{2T_1} e^{-2|\Delta t|/T_2} - \frac{1}{2T_1/T_2 - 1}\left(e^{-|\Delta t|/T_1} - e^{-2|\Delta t|/T_2}\right)\right], \tag{17}$$

where integration is carried out over all counts in the histogram bin at $\tau = 0$ (c.f. Supplementary Fig. 1). In Supplementary Fig. 3c and d, we plot Eq. 17 for the limiting cases considered above. In addition, we also include the case of NTD 1 (as a single source with non-unity single photon purity) probed in our experimental Mach-Zehnder interferometer with imperfect beamsplitters described in Supplementary Note 4. As explained above, in this case the values of $g^{(2)}_{\mathrm{HOM}}(\tau = 0)$ are offset due to interferometer imbalance and nonzero $g^{(2)}_{\mathrm{HBT}}(0)$.

As obvious from Supplementary Fig. 3c and d, the slow decay process will have an associated HOM timescale of 100 ps at two-photon interference visibility of around 0.1. By contrast, the fast decay process will have an associated HOM timescale of 2 ps, and unity interference visibility (HOM correlations do not vanish at $\Delta t = 0$ ps due to nonzero $g^{(2)}_{\mathrm{HBT}}(0)$). This motivates the interpretation presented in the main text: the fast decay process results in photons with near-unity visibility, which is reduced to our measured value of around 0.6 by photons generated at reduced indistinguishability via the slow process in the PL decay of Fig. 4e of the main text.

Finally, we note that for highly indistinguishable photons with $T_2 \approx 2T_1$, Eq. 17 simplifies to:

$$g^{(2)}_{\mathrm{HOM}}(\tau = 0) \approx \frac{1}{2}\left(1 - \frac{T_2}{2T_1} e^{-|\Delta t|/T_1}\right). \tag{18}$$

The function used to fit the data in Fig. 4d of the main text has the same functional form.

SUPPLEMENTARY NOTE 6 - EXPERIMENTAL CAVITY LINEWIDTH

The cavity linewidth κ was obtained from the cavity transmission of a diode laser with a wavelength measured as 1468.2 nm and limited by the resolution of the spectrometer. Supplementary Fig. 4a shows the measured transmission as the cavity length is tuned by 1.3 free spectral ranges (FSRs), obtained after laterally positioning the cavity mode on the bare mirror away from NTDs. The two resonances at maximum transmission correspond to the TEM_{00}-modes of the lowest accessible longitudinal mode orders $q = 4$ and 5, respectively. Their distance corresponds to exactly one FSR, or half the wavelength, and was used to compute the time-dependent change in cavity length. The additional resonances stem from higher order TEM modes. From Lorentzian fits to the transmission of the $q = 4$ resonance as in Supplementary Fig. 4b, we obtained an averaged cavity linewidth $\kappa = 35.4 \pm 0.1$ μeV from ten repetitions of the measurement.

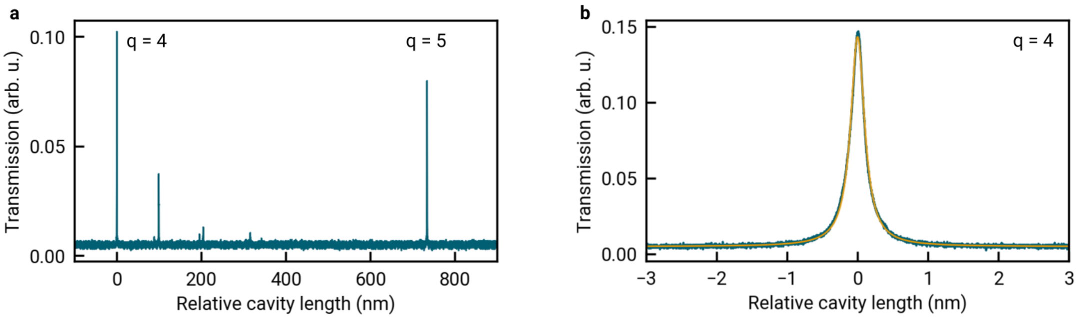

SUPPLEMENTARY FIG. 4. **Experimental cavity linewidth.** **a**, Cavity transmission at a wavelength of 1468.2 nm as a function of cavity length, which is tuned over 1.3 free spectral ranges **b**, Close-up on the resonance corresponding to longitudinal mode order $q = 4$ shown in **a** and best fit of a Lorentzian line profile (solid orange line) with a linewidth of $\kappa = 34.8$ μeV.

SUPPLEMENTARY REFERENCES

[1] Grange, T. *et al.* Cavity-Funneled Generation of Indistinguishable Single Photons from Strongly Dissipative Quantum Emitters. *Physical Review Letters* **114**, 193601 (2015).

[2] Hunger, D. *et al.* A fiber fabry–perot cavity with high finesse. *New Journal of Physics* **12**, 065038 (2010).

[3] He, X. *et al.* Tunable room-temperature single-photon emission at telecom wavelengths from sp3 defects in carbon nanotubes. *Nature Photonics* **11**, 577–582 (2017).

[4] Hartmann, N. F. *et al.* Photoluminescence Dynamics of Aryl sp3 Defect States in Single-Walled Carbon Nanotubes. *ACS Nano* **10**, 8355–8365 (2016).

[5] Auffèves, A., Gérard, J.-M. & Poizat, J.-P. Pure emitter dephasing: A resource for advanced solid-state single-photon sources. *Physical Review A* **79**, 053838 (2009).

[6] Auffèves, A. *et al.* Controlling the dynamics of a coupled atom-cavity system by pure dephasing. *Physical Review B* **81**, 245419 (2010).

[7] Jeantet, A. *et al.* Widely Tunable Single-Photon Source from a Carbon Nanotube in the Purcell Regime. *Physical Review Letters* **116**, 247402 (2016).

[8] Benedikter, J. *et al.* Cavity-Enhanced Single-Photon Source Based on the Silicon-Vacancy Center in Diamond. *Physical Review Applied* **7**, 024031 (2017).

[9] Purcell, E. Spontaneous emission probabilities at radio frequencies. *Phys. Rev.* **69**, 681 (1946).

[10] Kaupp, H. *et al.* Scaling laws of the cavity enhancement for nitrogen-vacancy centers in diamond. *Phys. Rev. A* **88**, 053812 (2013).

[11] Kiraz, A., Atatüre, M. & Imamoğlu, A. Quantum-dot single-photon sources: Prospects for applications in linear optics quantum-information processing. *Phys. Rev. A* **69**, 032305 (2004).

[12] Loredo, J. C. *et al.* Scalable performance in solid-state single-photon sources. *Optica* **3**, 433–440 (2016).

[13] Gokus, T. *et al.* Mono- and biexponential luminescence decays of individual single-walled carbon nanotubes. *The Journal of Physical Chemistry C* **114**, 14025–14028 (2010).

[14] Bylander, J., Robert-Philip, I. & Abram, I. Interference and correlation of two independent photons. *The European Physical Journal D - Atomic, Molecular, Optical and Plasma Physics* **22**, 295–301 (2003).

[15] Ollivier, H. *et al.* Hong-Ou-Mandel Interference with Imperfect Single Photon Sources. *Physical Review Letters* **126**, 063602 (2021).
